# Pathological and Molecular Characterization of *Avipoxvirus* Infection in *Burhinus oedicnemus* in the Canary Islands

**DOI:** 10.3390/vetsci12090849

**Published:** 2025-09-02

**Authors:** Ana Colom-Rivero, Antonio Fernández, Lucía Marrero-Ponce, Derke Padrón-Ramírez, Lucía Caballero-Hernández, Candela Rivero-Herrera, Cristian M. Suárez-Santana, Eva Sierra

**Affiliations:** 1 Unit of Veterinary Histology and Pathology, University Institute of Animal Health and Food Safety (IUSA), Veterinary School, University of Las Palmas de Gran Canaria (ULPGC), 35413 Las Palmas de Gran Canaria, Canary Islands, Spain; lucia.marrero@ulpgc.es (L.M.-P.); derkepr@gmail.com (D.P.-R.); lucia.caballero102@alu.ulpgc.es (L.C.-H.); candela.rivero101@alu.ulpgc.es (C.R.-H.); cristian.suarez@ulpgc.es (C.M.S.-S.); eva.sierra@ulpgc.es (E.S.); 2 Tafira Wildlife Rehabilatation Center (Cabildo de Gran Canaria), 35017 Las Palmas de Gran Canaria, Spain

**Keywords:** avian pox, Stone-curlew, *Aspergillus fumigatus*, poxvirus, squamous cell carcinoma

## Abstract

Avian poxvirus was diagnosed in eight wild Stone-curlews (*Burhinus oedicnemus*) from the Canary Islands, based on a combination of pathological and molecular analysis. Affected birds exhibited lesions consistent with poxvirus infection; three of four with mild lesions (≤2 on pelvic limbs, excluding phalanges) were successfully rehabilitated and released, while four with moderate (≤2 on phalanges) to severe lesions (≥3 on phalanges) potentially faced impaired mobility, increased predation risk, and reduced foraging efficiency. Histopathology of six individuals revealed Bollinger bodies, characteristic of *Avipoxvirus* infection, and molecular analysis confirmed the presence of viral DNA in six cases. Three genetically distinct viral variants were identified, each associated with different phylogenetic clades and subclades, suggesting substantial viral diversity within this host species. Co-infection with *Aspergillus fumigatus* was also detected in six of the eight birds, confirmed by molecular analysis in either skin lesions or lung tissue. To our knowledge, this represents the first report of *A. fumigatus* co-infection in Stone-curlews with *Avipoxvirus*. Additionally, one individual presented a tumor-like lesion, expanding the known pathological manifestations of the disease. These findings provide new insights into avian pox and highlight the importance of considering fungal co-infections in the differential diagnosis, given their potential to exacerbate disease severity.

## Simple Summary

Wild birds are vulnerable to diseases that can affect their health and survival. In this study, we examined eight Stone-curlews from the Canary Islands with signs of avian pox, a viral skin disease. Some birds had mild lesions and recovered with treatment, while others developed severe foot injuries that could hinder movement, feeding, and predator avoidance. Genetic analysis revealed different viral strains, showing high diversity within this host species. In addition to the virus, we detected skin fungal co-infections that may worsen the disease. Moreover, one bird presented an unusual tumor-like lesion, which has previously been described in association with avian pox in other species, expanding the range of known disease manifestations. These findings improve our understanding of health threats in wild birds and stress the need to consider multiple infections in wildlife disease research.

## 1. Introduction

Avian pox is a globally distributed infectious disease affecting birds caused by viruses of the genus *Avipoxvirus* (family *Poxviridae*) [1]. The International Committee on Taxonomy of Viruses (ICTV) currently recognizes 12 species within this genus, with two additional species recently proposed based on phylogenetic divergence and host range patterns [2]. Phylogenetic analyses of selected genomic regions have grouped *Avipoxvirus* strains into at least three major clades: fowlpox-like, canarypox-like, and psittacinepox-like [2,3,4]. However, despite this clade-level structure, species boundaries remain unresolved. A recent molecular survey identified 152 unique viral sequences, highlighting substantial genetic diversity within the genus [5], which has traditionally been characterized using a conserved 578 bp fragment of the 4b core protein gene [6], although broader genomic approaches are now increasingly applied.

Avian poxviruses infect a wide range of both domestic and wild avian species, with infections documented in more than 374 bird species spanning 23 avian orders, highlighting their broad host range [5]. However, while many viral lineages appear capable of infecting a broad range of host species across diverse avian orders, the extent of host specificity remains debated. Some strains show evidence of generalist behavior, potentially driven by anthropogenic factors, whereas others may display a more restricted, taxon-specific host range [3,7].

Clinically, avian pox manifests in three primary forms: cutaneous, diphtheritic, or mixed, with the cutaneous form being most prevalent [8]. This form typically involves nodular lesions on the comb, wattles, eyelids, and legs [9]. Disease severity varies depending on host susceptibility, viral strain virulence, location and number of injuries, and the presence of secondary infections or environmental stressors [9,10]. Although avian pox is typically self-limiting and associated with low mortality rates [11,12], severe outcomes can occur, particularly when secondary bacterial or fungal infections develop [13,14,15,16]. Such co-infections can exacerbate disease progression and may result in systemic illness [13,17].

The impact of Avipoxviruses on island bird populations is particularly concerning, with the virus implicated in population declines and potential local extinctions [18,19]. The Stone-curlew (*Burhinus oedicnemus*) is a ground-nesting bird of conservation concern in Europe. Genetic studies have revealed significant differentiation between mainland and island populations, with two endemic subspecies recognized in the Canary Islands: *B. o. insularum* (Fuerteventura and Lanzarote, including La Graciosa and Alegranza), and *B. o. distinctus* (Gran Canaria, Tenerife, La Gomera, El Hierro and La Palma) [20,21,22]. Despite its ecological and conservation significance, the susceptibility of this species to *Avipoxvirus* infection remains poorly understood [23,24,25].

This study seeks to explore the occurrence and genetic variability of *Avipoxvirus* in Stone-curlew populations, with special attention to the endemic subspecies of the Canary Islands. Through an integrated approach involving clinical examination, anatomopathological analysis, and molecular techniques, we aim to characterize the viral strains, determine their phylogenetic relationships, and enhance the understanding of avian pox ecology in wild bird species of conservation concern.

## 2. Materials and Methods

### 2.1. Animals and Tissue Sampling

This study includes eight Stone-curlews with cutaneous avian pox-like lesions, comprising three live cases and five necropsied individuals, conducted within the framework of the Canarian Wildlife Health Surveillance Network, a monitoring program on causes of mortality in Canarian wildlife established by the Canarian Government (Red Vigía Canarias; Order No. 134/2020, 26 May 2020). Of the eight individuals included in the present study, seven originated from Gran Canaria and one from Lanzarote (Table 1).

The prospective component included three Stone-curlews admitted to the Wildlife Rehabilitation Center of Tafira (WRCT) (Cabildo of Gran Canaria) between 2023 and 2024 with cutaneous lesions (on the pelvic limbs and/or beak) compatible with *Avipoxvirus* infection. Upon admission, all individuals underwent clinical examination, and when available for each specific case, skin biopsies and/or swabs were collected for further diagnostic investigation. These birds were monitored during the rehabilitation process and, following improvement in clinical symptoms and visible healing of cutaneous lesions, were subsequently released back into the wild (Table 1; Cases 2, 3, and 8).

The retrospective component involved the review of 68 Stone-curlews necropsied between 2021 and 2024 at the University Institute of Animal Health and Food Safety (Instituto Universitario de Sanidad Animal y Seguridad Alimentaria, IUSA), using standardized necropsy reports, routine histopathological evaluations, and macroscopic image analyses. Among these, five birds exhibited macroscopic cutaneous lesions suggestive of avian poxvirus infection, particularly affecting featherless areas of the pelvic limbs (legs and feet). These comprised one bird found dead in the field (Table 1; Case 1) and four admitted to the WRCT for veterinary care and potential release, of which two died naturally and two were humanely euthanized due to poor prognosis (Table 1; Cases 4–7).

Of the 8 Stone-curlews, 2 were identified as female and 3 as male; the sex of the remaining 3 could not be determined due to the condition of the carcasses or, in the case of live animals, the absence of external sexual dimorphism characteristic of this species. Age classification was based on external morphology, skeletal features, and gonadal development, and individuals were assigned as juvenile (*n* = 2) or adult (*n* = 4), while the age of 3 birds could not be determined. Nutritional status was assessed through visual and manual evaluation of the pectoral muscle mass (keel scoring) and the presence of fat reserves and classified on a five-point numerical scale, with 1 indicating cachexia, 2 underweight, 3 normal, 4 overweight, and 5 obese. This system represents a modification of the seven-category body condition scoring method described by Burton et al. (2014) [26]. In our approach, the category *cachexia* (score 1) was introduced to denote individuals with marked pectoral muscle atrophy, thereby distinguishing severe pathological muscle wasting from general underweight status. The categories *thin* and *lean* in Burton et al. were combined into a single *underweight* category (score 2), representing individuals without grossly appreciable muscle atrophy. *Ideal* corresponds to our *normal* (score 3), and *moderately overweight* corresponds to *overweight* (score 4). Finally, *severely overweight* and *morbidly overweight* were merged into a single category (*obese*, score 5), as these two categories did not reflect biologically or clinically meaningful differences in our study population of wild birds; both were characterized by excessive fat deposition that obscured body contours and masked pectoral muscle definition. Body condition assessments revealed that 3 were cachectic (grade 1) and 5 were underweight (grade 2). This case-specific information (including body weight) is summarized in Table 2.

Deceased Stone-curlews were stored under refrigeration (Cases 4 and 6) or frozen (Cases 1, 5, and 7) until necropsy (Table 2). Carcasses were subsequently classified according to their preservation status based on the degree of decomposition (1 = very fresh, 2 = fresh, and 3 = early decomposition), following standardized protocols established at the IUSA (Table 2).

All necropsies were performed in accordance with consistent and standardized procedures [27] and involved the systematic examination and photographic documentation of all organs and any observed lesions. During necropsy, tissue samples were collected from major organs, including the liver, lungs, kidneys, intestines, and any observed skin lesions (Table 2). Additionally, sterile, individually packaged swabs (Vircell S.L., Granada, Spain)—without transport medium—were used to collect samples from various body cavities, including the oropharyngeal cavity, cloaca, and coelomic cavity. Brain samples were also collected via this method as previously described [28,29]. All collected biological material was archived at the IUSA.

Frozen tissue samples from six individuals were processed for molecular analysis, and histopathological examination was performed on six cases (with partial overlap) from the same cohort of eight individuals (Table 2). All fresh, unfixed tissue and swab samples were stored at −80 °C until molecular virological analyses were conducted. Parallel tissue specimens were fixed in 4% buffered formalin for histopathological processing.

### 2.2. Gross and Histopathological Examination

During external evaluation, the number and anatomical distribution of visible hyperplastic, pox-like lesions were documented for each affected bird. Formalin-fixed tissue samples from each collected lesion were routinely processed, paraffin-embedded, sectioned at 3 μm, and stained with Hematoxylin and Eosin (H&E) for histopathological examination. The diagnosis of avian pox was based on the presence of characteristic lesions, including large, solid or ring-shaped eosinophilic intracytoplasmic inclusions (Bollinger bodies) within affected epithelial cells [9]. In cases with suspected secondary fungal infections, additional histochemical stain techniques, such as Grocott’s Methenamine Silver Nitrate (GMS) and/or Periodic acid Schiff (PAS) staining, were applied to assess the morphological characteristics of potential pathogens.

Lesion severity was evaluated based on anatomical location, lesion count, and the presence of secondary infections [1,12,16]. Given the terrestrial habits of Stone-curlews and the functional importance of their pelvic limbs in locomotion, lesions were classified as mild when limited to the pelvic limbs (excluding the phalanges) and numbering no more than two. Lesions were considered moderate if involving the phalanges but limited to two or fewer, and severe if affecting the phalanges with three or more lesions present.

### 2.3. Molecular Analysis

Cutaneous lesion samples, obtained through necropsies and biopsies, were processed by mechanical maceration of 0.025 g of tissue in 400 μL of DNA/RNA Shield™ (Zymo Research) using 2 mL ceramic bead Precellys^®^ tubes, followed by centrifugation. Two hundred microliters of each skin lesion macerate were used for simultaneous DNA and RNA extraction employing a magnetic bead-based method on an automated robotic platform, following the manufacturer’s instructions for the ZYMO DNA/RNA extraction kit (ZYMO Research, Freiburg, Germany). To ensure the accuracy and reliability of the extraction process, both a negative control (nuclease-free water) and a positive control (a herpesvirus-positive sample previously confirmed in our laboratory) were included in each extraction batch [29].

The presence of the *Avipoxvirus* (AVP)-DNA was assessed in 10 samples corresponding to 9 skin lesions and 1 beak lesion. For the detection of viral DNA, a real-time semiquantitative PCR (sq-PCR) assay targeting the P4b gen, which encodes the conserved structural 4b core protein, was performed, as previously described [30]. The primer pair vAAPV-124f (5′-ACGTCAACTCATGACTGGCAAT-3′) and vAAPV-246r (5′-TCTCATAACTCGAATAAGATCTTGTATCG-3′) was used along with an internal hydrolysis probe vAAPV-159p-(5′-FAM-AGACGCAGACGCTATA-MGB-3′) labelled with a 5′ reporter dye (FAM) and a 3′ quencher (Non-Fluorescent Quencher Minor Grove Binding, NFQMGB). Subsequently, all samples were subjected to a conventional PCR protocol to amplify a longer 578 bp of the same gene, as previously described [31]. The primers used were P1 (5′-CAGCAGGTGCTAAACAACAA-3′) and P2 (5′-CGGTAGCTTAACGCCGAATA-3′), enabling downstream sequence analysis for species identification. PCR products (5 μL) were analyzed by horizontal electrophoresis on a 2% agarose gel containing GelRed^®^ (Biotium, Inc., Fremont, CA, USA). Diethylpyrocarbonate (DPEC)-treated water was used as the negative control for both PCRs, while an AVP-positive sample previously confirmed in our laboratory served as the positive control.

In this study, the definitive molecular status of cutaneous lesion samples for *Avipoxvirus* (positive or negative) was determined by the combined results of all molecular assays performed, including both conventional and real-time PCR techniques. A sample was classified as positive if at least one of the PCR methods yielded a positive result. These molecular findings were further interpreted in conjunction with the histopathological evaluation of the corresponding formalin-fixed paraffin-embedded tissue samples.

In one case involving suspected secondary fungal infection, lung, liver, and kidney tissues were directly pooled into a composite sample using a sterile swab. The swab was then transferred into a tube containing viral transport medium (VTM) (Transport Medium for the Collection and Preservation of Viruses, Chlamydia, and Mycoplasma, Vircell S.L.), which contains HEPES buffer, gelatine, bovine serum albumin, sucrose, and compatible antibiotics to ensure pathogen stability and viability during both transport and storage. The sample was thoroughly mixed, and 100 μL of VTM was combined with an equal volume of DNA/RNA Shield™ (Zymo Research, Freiburg, Germany) prior to nucleic acid extraction, using the same magnetic bead-based automated method described above.

To confirm the presence of fungal infection, specifically *Aspergillus fumigatus*, a sq-PCR assay was performed using the primer pair Af25S-F (5’-GGGTCGAACGGTCAAGT-3’) and Af25S-R (5’-GAGAGTCCATGGAGGTGGAG –3’), which amplifies a 202 bp fragment of the 25S rRNA gene based on the GenBank sequence U15421. The sq-PCR was conducted using SYBR Green chemistry (Bio-Rad), following the manufacturer’s recommended protocols.

### 2.4. Detection, Sequencing of PCR Products, and Phylogenetic Analysis

Conventional PCR amplicons from AVP-DNA-positive samples were purified using the Real Clean spin kit (REAL, Valencia, Spain) and subsequently subjected to bidirectional Sanger sequencing. The resulting nucleotide sequences were analyzed and compared both internally and with publicly available sequences in GenBank through the BLAST 2.15.0 (Basic Local Alignment Search Tool, (https://blast.ncbi.nlm.nih.gov/Blast.cgi, accessed on 22 October 2023) algorithm. Multiple sequence alignments of AVP sequences were carried out using ClustalW. The resulting nucleotide sequences were deposited in GenBank (accession numbers: PV976817, PV976818, PV976819, PV976820 and PV976821).

Phylogenetic and molecular evolutionary analyses were performed using MEGA version 12 [32], incorporating 41 AVP nucleotide sequences retrieved from GenBank. Phylogenetic trees were constructed using the Neighbor-Joining (NJ) and BioNJ algorithms based on pairwise genetic distances estimated by the Maximum Composite Likelihood (MCL) method under the Tamura 3 parameter (1985) substitution model Nei & Kumar, 2000. Rate variations among sites were modelled using a discrete Gamma distribution with 5 categories (+G, shape parameter = 0.3839). The analysis included 45 nucleotide sequences, with 1050 aligned positions in the final dataset. The topology robustness was assessed using a bootstrap consensus tree based on 1000 replicates. Branches supported by fewer than 50% of bootstrap replicates were collapsed, and bootstrap values (expressed as percentages) were displayed next to the corresponding branches [33].

## 3. Results

### 3.1. Gross and Histopathological Findings

**Figure 1:**
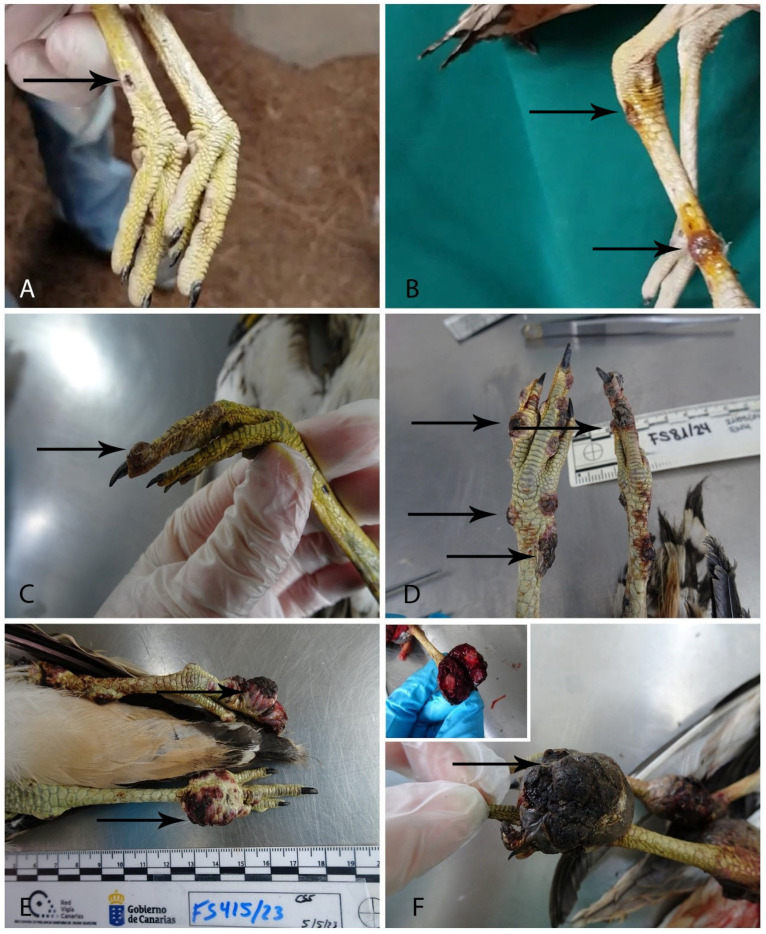
Gross cutaneous manifestations of *Avipoxvirus* infection in the Stone-curlews included in this study, presented in increasing severity from mild to severe. (**A**). Case 3. Mild. Small vesicular lesion on the tarsometatarsus (arrow). (**B**). Case 2. Mild. Multiple pink ulcerative and vesicular lesions on the tarsometatarsus, treated with iodine at the WRCT (arrows). (**C**) Case 6. Moderate. One vesicular lesion on the phalanges (arrow). (**D**). Case 5. Severe. Multiple red nodular lesions on the leg tarsometatarsus and phalanges (arrows). (**E**). Case 4. Severe. Multiple red eroded nodules on the tarsometatarsus and phalanges (arrows). (**F**). Case 7. Severe. A large, ulcerated nodule occupying nearly the entire phalangeal surface (arrow).

**Figure 2:**
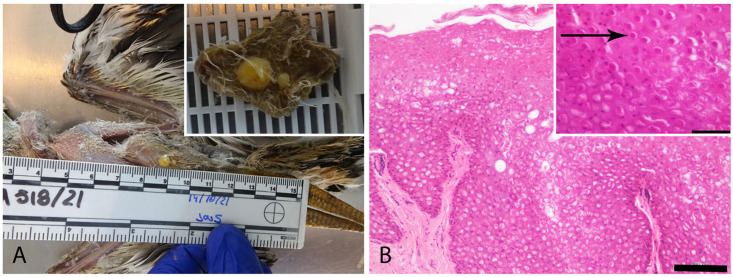
Characteristics of the pox-like lesion in Case 1 (**A**). Gross presentation of the cutaneous yellow form of *Avipoxvirus* infection, characterized by two yellowish contiguous nodular lesions on the skin. (**B**). Histopathological section showing characteristic epidermal hyperplasia, ballooning degeneration of keratinocytes, and the presence of solid intracytoplasmic inclusion bodies indicative of *Avipoxvirus* infection (Bollinger bodies: BB) (Bar = 100 µm). Inset: Higher magnification of the BB (arrow) (Bar = 50 µm).

Gross examination of the eight animals from our study revealed multiple raised, wart-like cutaneous vesicles or nodules, varying in color, typically pink, red (Figure 1), or yellow, as observed in Case 1 (Figure 2A). These lesions were predominantly located on the pelvic limbs, including the area of the tibiotarsus, the tarsometatarsus, and phalanges. Lesion numbers varied among individuals, ranging from two to more than three per animal, and displayed diverse morphologies and surface characteristics, from a small, smooth, yellowish nodule, to multiple ulcerated, coalescing, nodules (Table 3) (Figure 1 and Figure 2A).

**Figure 3:**
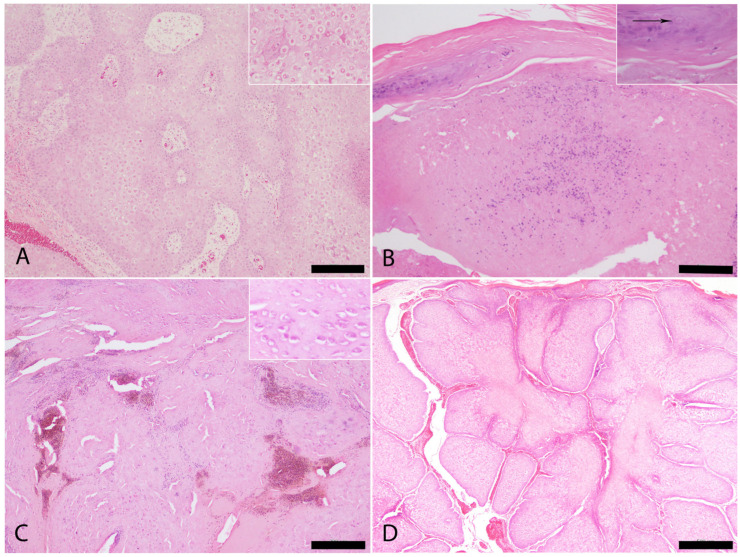
Histopathological findings related to *Avipoxvirus* infection. Hematoxylin and Eosin stain. (**A**). Case 2. Severe epidermal hyperplasia with ring-like eosinophilic intracytoplasmic inclusion bodies (Bollinger bodies) (Bar = 200 µm). Inset: higher magnification of the BB. (**B**). Case 6. Extensive areas of necrosis and heterophilic inflammation, accompanied by mild epidermal hyperplasia and scattered solid eosinophilic intracytoplasmic inclusion body-like inclusions (Bar = 200 µm). Inset: higher magnification of the intracytoplasmic inclusion body-like inclusions (arrow). (**C**). Case 7. Marked epidermal hyperplasia with the presence of solid eosinophilic intracytoplasmic inclusion bodies (BB) in keratinocytes (Bar = 200 µm). Inset: higher magnification of the BB. (**D**). Case 4. Pronounced epidermal hyperplasia featuring characteristic eosinophilic intracytoplasmic inclusion bodies (BB) and central areas of necrosis (Bar = 500 µm).

Histopathological examination of six of the eight cases from our study showed focal to multifocal epidermal thickening due to keratinocyte hyperplasia, particularly within the stratum spinosum, the presence of characteristic eosinophilic intracytoplasmic inclusion bodies (Bollinger bodies) in the infected keratinocytes (solid or ring-shaped), and variable degrees of epidermal necrosis (Figure 2B and Figure 3). Additional histopathological findings included heterophilic inflammatory infiltrates in five of six cases (83.3%), ballooning degeneration in five of six (83.3%), hemorrhage in four of six (66.7%), erosion in four of six (66.7%), and ulceration in four of six (66.7%) (Figure 3). Keratin pearls were identified exclusively in two cases (Case 2 and Case 4) (Figure 3). Histopathological evaluation could not be performed for Cases 3 and 8 due to the unavailability of biopsy material.

**Figure 4:**
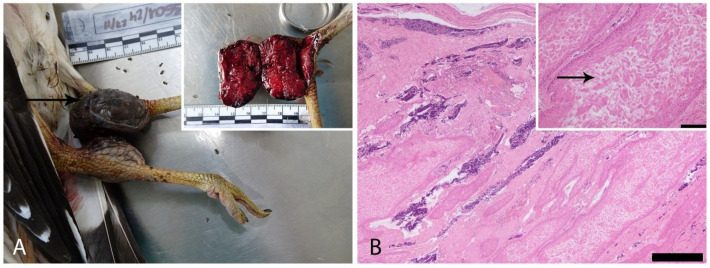
Tumor-like lesions compatible with a squamous cell carcinoma associated to *Avipoxvirus* infection in Case 7. (**A**). Gross finding. Tumor-like lesions identified in Case 7. (**B**). Histopathological changes consistent with irregular infiltrative cords of keratinocytes (Bar = 200 µm). Inset: squamous differentiation of proliferative and invasive keratinocytes (arrow), compatible with a squamous cell carcinoma (Bar = 100 µm).

Tumor-like proliferative lesions associated with cutaneous *Avipoxvirus* infection were identified in Case 7 (Figure 4A). Histologically, adjacent to areas of marked epithelial hyperplasia with Bollinger bodies (Figure 3C), an invasive proliferation was observed. It was composed of irregular infiltrative cords of proliferating keratinocytes and nests of atypical epithelial cells with prominent squamous differentiation. Other histological features included keratinization, intercellular bridges, and nuclear pleomorphism, consistent with a diagnosis of locally invasive squamous cell carcinoma (Figure 4B).

**Figure 5:**
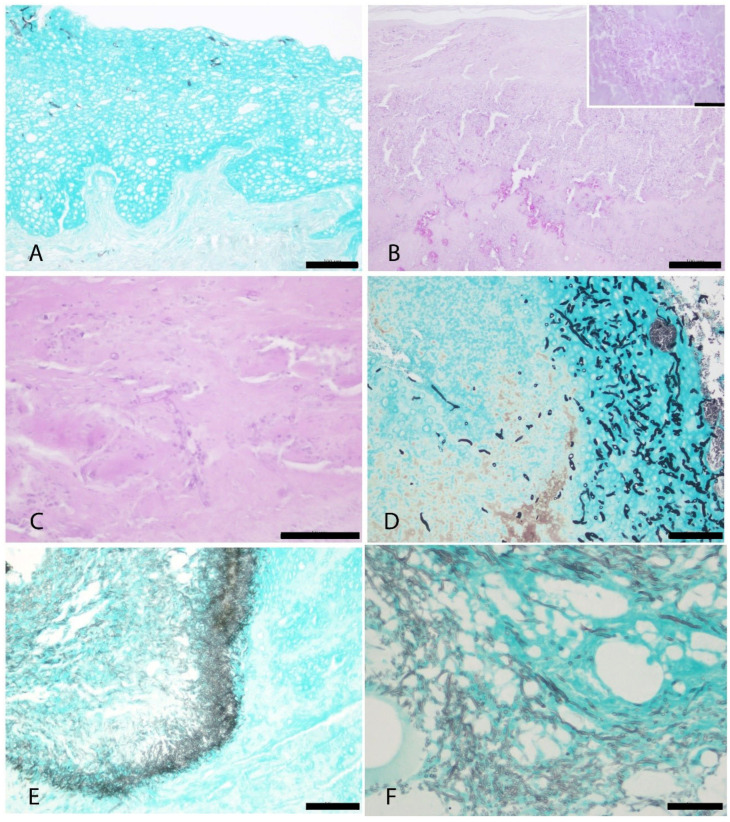
Histopathological findings of secondary fungal co-infections. (**A**). Case 1. Fungal hyphae were observed in the superficial stratum of the epidermis, without associated histopathological alterations. GMS stain (Bar = 100 µm). (**B**). Case 6. Fungal hyphae are present in middle layers of the hyperplastic epidermis, accompanied by necrosis and inflammation. PAS stain (Bar = 100 µm). Inset: higher magnification of the intralesional hyphae. PAS stain (Bar = 50 µm). (**C**). Case 4. Branched, parallel, dichotomous hyphae, consistent with *Aspergillus fumigatus*, are embedded within a necrotic and inflamed area of the hyperplastic epidermis. PAS stain (Bar = 50 µm). (**D**). Case 2. Infiltrating and invasive fungal hyphae were observed within the proliferative epithelium. GMS stain (Bar = 50 µm). (**E**). Case 7, skin. Short, thin septate fungal hyphae stained black, with angular and dichotomous branching, suggestive of *Aspergillus* sp. (Bar = 50 µm). (**F**). Case 7, lung. Similar fungal hyphae observed, consistent with *Aspergillus* infection, confirmed by PCR as *A. fumigatus* (Bar = 50 µm).

Regarding co-infections, superficial and/or intralesional bacteria were observed in four of the six animals evaluated histologically. Additionally, 3.5 to 4.5 μm, tubular, septate hyphae, with parallel walls, and dichotomous branching, morphologically consistent with *Aspergillus* spp. were identified in cutaneous lesions from five cases (Cases 1, 2, 4, 6, and 7), as well as in the lung tissue of Case 7 (Figure 5) (Table 3).

### 3.2. PCR and Sequence Analyses

**Figure 6:**
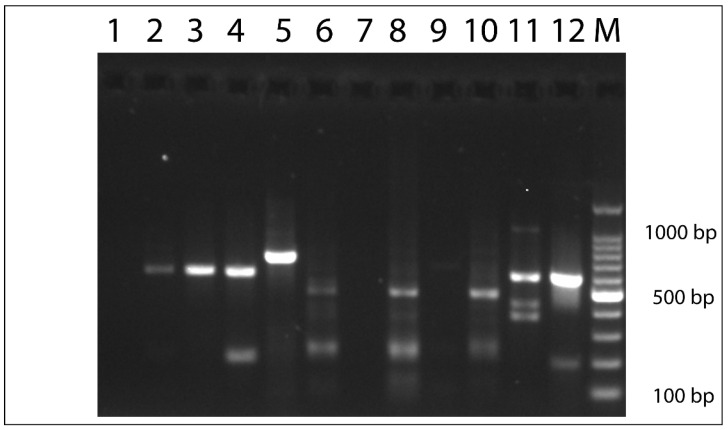
Agarose gel (2%) electrophoresis of conventional PCR products. (A). Specific AVP-DNA amplified products (~578 bp) are detected in lane 2 (Case 2), lane 3 (Case 3), lane 4 (Case 7), lane 11 (Case 8), and lane 12 (Case 4 and our PCR positive control). Last lane (M) contains a 100 bp DNA ladder.

*Avipoxvirus* P4b DNA was detected in skin lesion samples from all six birds analyzed by molecular methods. Of the ten samples tested, eight (exclusively obtained from the pelvic limbs) were positive (Table 4). All positive samples yielded amplification with both conventional and sq-PCR assays, except in two cases (Table 3), in which amplification was detected using only one of the two assays. The AVP-DNA positive samples were further evaluated based on their cycle threshold (Ct) values, with amplification beyond a Ct of 35 considered negative by the sq-PCR criteria. In Case 6, only the sq-PCR assay produced a positive result, while the conventional PCR failed to generate an amplicon of the expected size. In contrast, in Case 7, no amplification was observed by sq-PCR; however, a distinct band of 578 bp, corresponding to the expected product of the conventional PCR, was visualized on the 2% agarose gel (Figure 6) (Table 4).

Fresh frozen tissue samples were not available for Case 1 and Case 5, precluding confirmation of viral presence through PCR-based methods. Amplicons obtained from conventional PCR (Cases 2, 3, 4, 7, and 8) and the sq-PCR product from Case 6 were successfully sequenced. BLAST analysis of the conventional PCR amplicons revealed varying degrees of homology between the P4b gene sequences from each bird and previously described AVP sequences. These sequences, with 100% coverage, showed identities ranging from 100% to 97.05%, depending on the AVP species.

DNA of *Aspergillus fumigatus* was detected in five of the eight birds analyzed, supporting the histopathological findings. An exception was noted in the skin sample from Case 7, which showed fungal elements upon microscopic examination (Figure 5) but yielded a negative PCR result. However, the corresponding pooled sample, which included lung tissue, tested positive.

### 3.3. Phylogenetic Analysis

**Figure 7:**
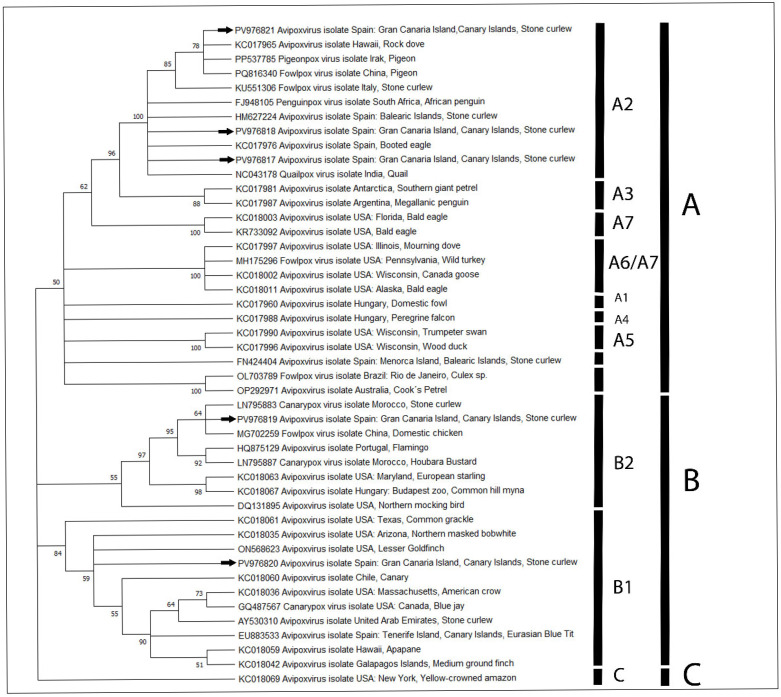
Phylogenetic analysis of cases 2, 3, 4, 7, and 8 AVP-positive (arrow) (GenBank accession numbers: PV976817, PV976818, PV976819, PV976820, and PV976821), obtained from skin samples, based on partial sequences of the P4b gene from different host species. Clades A, B, and C (and their subclades, see Gyuranecz et al., 2013 [7]) are labelled. The evolutionary history was inferred by using the Maximum Likelihood method [33]. The bootstrap consensus tree inferred from 1000 replicates [35] is taken to represent the evolutionary history of the taxa analyzed. Branches corresponding to partitions reproduced in less than 50% bootstrap replicates are collapsed. The percentages of replicate trees in which the associated taxa clustered together in the bootstrap test (1000 replicates are shown next to the branches [35]. Initial tree(s) for the heuristic search were obtained automatically by applying Neighbor-Joining [36] and BioNJ [37] algorithms to a matrix of pairwise distances estimated using the Maximum Composite Likelihood (MCL) [33] approach and then selecting the topology with superior log likelihood value. A discrete Gamma distribution across 5 categories (+G, parameter = 0.3839). The analytical procedure encompassed 46 nucleotide sequences with 1.050 positions in the final dataset. Evolutionary analyses were conducted in MEGA12 [32].

The P4b gene nucleotide sequences obtained from six AVP-positive animals were analyzed to evaluate their phylogenetic relationships. A fragment of approximately 578 bp from each P4b sequence was used to construct a phylogenetic tree, incorporating 41 reference AVP sequences representing the three major clades. Phylogenetic inference, performed using the T92 + G substitution model (Figure 7), placed the newly generated sequences within the established AVP clades, as previously described by Gyuranecz et al. (2013), Jarmin et al. (2006), and McInnes et al. (2023) [2,3,7]. The tree showed strong bootstrap support for the branching pattern. The AVP sequences from this study were distributed among distinct clades: three cases grouped within clade A2 (100%), clustering with two previously identified AVP sequences from Stone-curlews (HM627224 [34], KU551306 [24]); Case 4 was assigned to clade B2 (87%); and Case 5 was grouped within clade B1 (84%), together with another AVP-DNA sequence previously reported in a Stone-curlew (AY530310 [6]) (Figure 7).

## 4. Discussion

The Stone-curlew is a migratory species of conservation concern, included in Annex I of the EU Birds Directive. *Avipoxvirus* infections have been reported in both wild and captive populations of this species across various regions, with a notably high prevalence in island environments like the Canary Islands, Balearic Islands, and Sardinia. Consistent with our findings, these infections frequently manifest as cutaneous lesions on the legs [6,23,24,25,34].

Avian pox was diagnosed in 8 out of 71 (11.3%) Stone-curlews examined, which included 68 deceased individuals and three that were rehabilitated and subsequently released into the wild following a period of recovery at a wildlife rehabilitation center (WRCT). Of the eight individuals exhibiting gross lesions suggestive of poxvirus infection, six underwent histopathological examination, all of which showed features consistent with *Avipoxvirus* infection. Molecular confirmation (detection of AVP-DNA) was achieved in four of these six cases. Additionally, AVP-DNA was detected in two lesion samples from individuals that were released and therefore not subjected to histopathological evaluation, as biopsies were only collected for molecular confirmation of the infection.

Our study represents the first systematic investigation of avian pox in wild Stone-curlews, integrating gross, histopathological, and molecular analyses from a collection of affected individuals. Prior reports of *Avipoxvirus* infection in this species have been largely limited to anecdotal or isolated cases. For instance, in the Canary Islands, five wild Stone-curlews admitted to a wildlife rehabilitation center (WRCT) showed gross lesions, with electron microscopy confirming the diagnosis in two cases but lacking molecular analysis [25]. Other documented cases include a wild Stone-curlew from Sardinia with both pathological and molecular confirmation of APV infection by PCR [24], and molecular detection from two specimens collected in the Balearic Islands in 1980 [34] and in Morocco in 2013 [38], respectively, although pathological descriptions were not provided for these latter cases. Clinical descriptions, together with pathological and molecular analysis, have also been reported in captive, purchased individuals, which likely became infected via cross-contamination with a wild passerine bird introduced into the same farm [23]. In contrast, the Stone-curlews in our study became infected in their natural habitat, representing the first comprehensive characterization of naturally occurring avian pox in a cohort of wild individuals.

Macroscopic and microscopic examination of eight *Avipoxvirus*-infected Stone-curlews in this study revealed no evidence of systemic involvement, supporting their classification as localized, non-generalized cases of the dry cutaneous form of avian pox. Lesions predominantly affected featherless areas of the pelvic limbs (legs and toes) and were either proliferative and/or erosive-ulcerative. However, in one case, two lesions were also observed on a feathered region of the thigh, presenting as prominent, smooth, yellow nodules, a manifestation that although occasionally reported in avian pox infections, is not considered typical [9]. Based on lesion count and anatomical distribution, the majority of cases were classified as minimal (50%), whereas moderate (12.5%) and severe (37.5%) lesions together comprised the same proportion (50%). Mild infections typically present as small lesions (1–5 mm) and are generally non-debilitating [34]. Notably, three of the four cases classified as minimal involved animals that were rehabilitated and subsequently released into the wild without long-term field follow-up, thereby limiting our understanding of the risk of disease recurrence or potential long-term impacts on the local population. This observation is consistent with the self-limiting nature of the infection in immunocompetent individuals, although veterinary intervention may have contributed to lesion resolution and the overall favorable clinical outcome observed in these individuals. Conversely, larger lesions, as observed in three individuals and primarily affecting the phalangeal region, covering more than 10% of the surface area, were classified as severe in our study due to their potential to significantly impair locomotor function [5,39]. In ground-nesting, steppe-associated species such as the Stone-curlew, reduced mobility due to extensive cutaneous lesions may heighten susceptibility to predation and hinder foraging efficiency, thereby indirectly contributing to increased mortality risk [1,20,21,25].

Moreover, although the cutaneous form of avian pox is rarely a direct cause of death, it can negatively impact both survival and reproductive success by predisposing affected individuals to secondary infections [5]. Opportunistic bacterial and fungal pathogens are among the most frequently reported complications associated with the cutaneous form of avian pox, contributing significantly to disease severity and clinical outcome [40]. In the present study, co-infections with fungal pathogens were detected in nearly all examined individuals, and bacterial co-infections were also observed in several cases, highlighting the role of opportunistic infections in disease progression and clinical outcome. Although bacterial agents could not be identified at the genus or species level, their detection in skin tissue samples indicates a possible contribution to the observed pathology. Nevertheless, the absence of taxonomic resolution hampers our understanding of their ecological role and their interactions with the primary infection. Further investigation is required to elucidate these relationships. In contrast, fungal pathogens were more clearly characterized, with *Aspergillus fumigatus* molecularly confirmed in skin lesions or lung tissue in six out of eight Stone-curlews affected by avian pox. To our knowledge, this is the first documentation of this co-infection in Stone-curlews, although similar dual infections have been reported in other avian species [14,17]. The cutaneous lesions associated with avian pox may have facilitated secondary fungal invasion, as previously suggested for other fungal species like *Aspergillus* spp. and *Candida* spp. [16]. *A. fumigatus* is a widespread airborne fungus that causes aspergillosis, a common respiratory disease in birds, as it was observed in one Stone-curlew from our study, particularly when immunity is compromised [41]. Our findings highlight the importance of considering fungal co-infections, particularly with *A. fumigatus*, in the evaluation of avian pox cases, especially when respiratory or systemic signs are present.

Additionally, tumor-like lesions were observed in one individual infected with *Avipoxvirus*. Histopathological examination revealed features resembling squamous cell carcinoma, a neoplastic condition that has been associated with avian poxvirus infections in previous studies [42,43]. Some authors have suggested that chronic *Avipoxvirus*-induced epithelial proliferation and inflammation may contribute to neoplastic transformation, although the causal relationship remains unclear [9,44].

This study reveals notable molecular variability among *Avipoxvirus* strains infecting two Stone-curlew subspecies in the Canary Islands, while prior research had only confirmed *Avipoxvirus* infection in the subspecies *B. o. distinctus* via electron microscopy [25]. Our analysis identified three distinct viral variants in just six APV-DNA-positive individuals. These variants were associated with different phylogenetic clades and subclades, indicating potentially high genetic diversity within APV strains affecting this host species. This diversity likely reflects a combination of ecological and epidemiological factors, including vector diversity, interspecies transmission, and anthropogenic movement of birds [9]. Different insect vectors can carry distinct viral strains [8,45,46], while sympatric bird species and human-mediated translocations may facilitate cross-species spillover and local mixing of variants [7,45]. Such mechanisms are consistent with previous observations of high *Avipoxvirus* diversity within single host species across localized populations [47]. Nonetheless, the presence of several low-support branches (collapsed at the 50% bootstrap threshold) in our analysis suggests that alternative phylogenetic topologies cannot be ruled out, and the inferred relationships among these variants should therefore be interpreted cautiously. Within this context of limited confidence, the observed clustering remains broadly consistent with previous molecular studies that revealed a remarkable genomic diversity among Avipoxviruses, surpassing the number of species currently recognized by the ICTV, and highlighted the capacity of several strains to infect multiple avian hosts [5]. Recent phylogenetic analyses have demonstrated that the divergence between APV clades is sometimes comparable to, or even exceeds, that observed among mammalian poxvirus genera. This has led to calls for a taxonomic reassessment of the genus *Avipoxvirus* [2].

The detection of *Avipoxvirus* in endemic Stone-curlew subspecies populations raises important conservation concerns, not only for this species but also for other vulnerable and range-restricted avifauna in the Canary Islands. Historical epizootic events in island ecosystems, including those in Hawaii, the Galápagos, and the Canary Islands, have demonstrated the highly invasive and pathogenic nature of Avipoxviruses in immunologically naive bird populations, with infection prevalence reaching up to 88% and contributing to population declines in several endemic taxa, including finches, pipits, larks, and albatrosses [19,25,48]. In this context, our study provides a detailed pathological and genetic characterization of avian poxviruses infecting wild Stone-curlews in the Canary Islands during a 4-year period, offering a comprehensive overview and a more complete understanding of the pathology, distribution, and diagnostic features of avian pox in this island-endemic and endangered population. We systematically documented lesions, confirmed infection through histopathological features consistent with poxvirus, and detected AVP-DNA using both conventional and semi-quantitative PCR. By integrating gross, histopathological, and molecular analyses across multiple natural cases of infection, our findings contribute novel and systematic information that was previously lacking in this species, including the detection of *A. fumigatus* co-infection and the presence of a tumor-like lesion associated with avian pox. However, a limitation of this study was the absence of molecular confirmation in two cases and the inability to perform histopathological assessment in another two. These constraints emphasize the need for standardized diagnostic approaches, as consistent use of molecular and histopathological techniques would enhance comparability, strengthen analyses, and improve result interpretation, especially in rare species or small-sample studies.

This infectious disease may represent a potential threat to the conservation of endemic Stone-curlew populations within the archipelago. Although infectious diseases are less frequently listed as primary drivers of extinction relative to habitat loss or overexploitation, their role in population declines should not be underestimated. According to the IUCN Red List, infectious diseases have been implicated in approximately 3.7% of documented vertebrate extinctions over the past five centuries [18], underscoring the importance of integrating disease surveillance and management into conservation planning for susceptible species such as the Stone-curlew.

## Figures and Tables

**Table 1:** Case information for Stone-curlews (*Burhinus oedicnemus*) included in this study, summarizing case number, internal identification code, status at discovery in the field, finding location and date, admission to the WRCT (when applicable), rehabilitation history, clinical outcome, and sample origin.

Case Code	ID Ref.	Status		FL	FD	Admission at WRCT		Days at WRCT	Clinical Outcome at WRCT			Sample Origin	
		Dead	Alive			Yes	No		Died	Euthanized	Released	Necropsy	Biopsy
1	SA518/21	X	-	L	06/04/21	-	X	NA	NA	NA	NA	X	-
2	123/23	-	X	GC	08/03/23	X	-	74	-	-	X	-	X
3	237/23	-	X	GC	14/04/23	X	-	45	-	-	X	-	X
4	FS415/23	-	X	GC	04/05/23	X	-	<1 (few hours)	-	X	-	X	-
5	FS81/24	-	X	GC	29/07/23	X	-	11	X	-	-	X	-
6	FS499/24	-	X	GC	03/09/24	X	-	1	X	-	-	X	-
7	FS601/24	-	X	GC	25/06/24	X	-	4	-	X	-	X	-
8	757/24	-	X	GC	18/08/24	X	-	44	-	-	X	-	X
**Total**	8	1	7	-	-	7	1	-	2	2	3	5	3

**Notes:** FL (finding location: GC = Gran Canaria; L = Lanzarote); FD (finding date); WRCT = Wildlife Rehabilitation Center of the Cabildo of Gran Canaria; NA = not applicable.

**Table 2:** Summary data from eight Stone-curlews (*Burhinus oedicnemus*) included in the present study (2021–2024), comprising biological information (age and sex), carcass preservation status at necropsy and decomposition state at necropsy (when applicable), body weight, body condition, and samples analyzed for *Avipoxvirus* by histopathology and DNA detection.

Case Code	MP	DC	W	BC	AGE	SEX	Cutaneous Lesion Tested	Histopathology	Molecular Testing
1	F	2	145.9	2	J	M	NA	Yes	No
2	NA	NA	295	2	U	U	Beak Skin 1 Skin 2	Yes	Yes
3	NA	NA	320	2	A	U	Skin 1 Skin 2 Skin 3	No	Yes
4	R	1	213.9	1	A	F	Skin	Yes	Yes
5	F	3	174.5	2	J	F	NA	Yes	No
6	R	2	275.6	1	A	M	Skin	Yes	Yes
7	F	2	212.8	1	A	M	Skin	Yes	Yes
8	NA	NA	310	2	U	U	Skin	No	Yes

**Notes:** DC = decomposition code: (1 = very fresh, 2 = fresh, 3 = incipient decomposition); MP = method of carcass preservation (F = freezing; R = refrigeration); W = weight; BC = body condition: (1 = cachexia, 2 = thin (slim); AGE (A = adult, J = juvenile, U = undetermined); SEX (F = female, M = male, U = undetermined); NA = not available or not applicable.

**Table 3:** Summary of gross and histopathological characteristics of cutaneous lesions observed in the eight animals included in this study.

Case Code	Gross Findings			Histopathology												
	L	NI	S	TS	BB	HP	HQ	BD	II	N	E	U	H	KP	F	B
**1**	T	2	Mild	Skin	+	+	-	+	+	+	-	-	-	-	+	-
**2**	TM Beak	2 1	Mild	Skin	+	+	+	+	+	+	+	+	+	+	+	+
				Skin	NA	NA	NA	NA	NA	NA	NA	NA	NA	NA	NA	NA
**3**	TM	2	Mild	Skin	NA	NA	NA	NA	NA	NA	NA	NA	NA	NA	NA	NA
**4**	TM, PH	+3	Severe	Skin	+	+	+	+	+	+	+	+	+	+	+	+
**5**	TM, PH	+3	Severe	Skin	+	+	-	+	+	+	+	+	+	-	-	+
**6**	PH	2	Moderate	Skin	+	+	+	-	+	+	-	-	-	-	+	-
**7**	TM, PH	+3	Severe	Skin	+	+	-	+	+	+	+	+	+	-	+	+
**8**	TM	2	Mild	Skin	NA	NA	NA	NA	NA	NA	NA	NA	NA	NA	NA	NA

**Note**: Gross findings [L = Location (T = Tibiotarsus; TM = Tarsometatarsus; PH = Phalanges); NI = Number of lesions; S = Severity of lesions]; Histopathology [(TS = Tissue Sample; BB = Bollinger bodies; HP = hyperplasia of the epithelium; HQ = hyperkeratosis; BD = ballooning degeneration of keratinocytes; II = inflammatory infiltrates; N = necrosis; E = erosion; U = ulceration; H = hemorrhage; KP = keratin pearls; F = Fungus; B = Bacteria); (+ = presence; - = absence; NA = Not available, not applicable)].

**Table 4:** Results of the PCR methods for the detection of AVP-DNA and *Aspergillus fumigatus*.

Case Code	Source for DNA Extraction	Sq-RT-PCR (123 bp)	Ct	Conventional PCR (587 bp)	Sq	Sq-RT-PCR *Aspergillus fumigatus* Results/Ct
**1**	Skin	NA	NA	NA	NA	NA
**2**	Beak	-	NA	-	NA	-
	Skin 1	+	30.03	+	Yes	-
	Skin 2	+	20.90	+	Yes	- 30.80
**3**	Skin 1	-	NA	-	NA	-
	Skin 2	+	28.25	+	Yes	+ 31.13
	Skin 3	+	24.98	+	Yes	+ 31.73
**4**	Skin	+	30.32	+	Yes	+ 36.44
**5**	NA	NA	NA	NA	NA	NA
**6**	Skin	+	29.53	-	NA	+ 32.06
**7**	Skin	-	NA	+	Yes	-
	Pool *	NA	NA	NA	NA	+ 33.28
**8**	Skin	+	23.48	+	Yes	-

**Notes:** Ct = cycle threshold; NA = not applicable, not available; Sq = sequencing.

## Data Availability

Data are available upon request.

## Abbreviations

The following abbreviations are used in this manuscript:

ICTV	International Committee on Taxonomy of Viruses
WRCT	Wildlife Rehabilitation Center of Tafira (Cabildo of Gran Canaria)
AVP	Avipoxvirus

## References

[1] Smits J.E., Tella J.L., Carrete M., Serrano D., López G. (2005). An epizootic of avian pox in endemic short-toed larks (*Calandrella rufescens*) and Berthelot’s pipits (*Anthus berthelotti*) in the Canary Islands, Spain. Vet. Pathol..

[2] McInnes C.J., Damon I.K., Smith G.L., McFadden G., Isaacs S.N., Roper R.L., Evans D.H., Damaso C.R., Carulei O., Wise L.M. (2023). ICTV Virus Taxonomy Profile: Poxviridae 2023. J. Gen. Virol..

[3] Jarmin S., Manvell R., Gough R.E., Laidlaw S.M., Skinner M.A. (2006). Avipoxvirus phylogenetics: Identification of a PCR length polymorphism that discriminates between the two major clades. J. Gen. Virol..

[4] Boyle D.B. (2007). Genus *Avipoxvirus*. Poxviruses.

[5] Williams R.A.J., Truchado D.A., Benitez L. (2021). A review on the prevalence of poxvirus disease in free-living and captive wild birds. Microbiol. Res..

[6] Lüschow D., Hoffmann T., Hafez H.M. (2004). Differentiation of avian poxvirus strains on the basis of nucleotide sequences of 4b gene fragment. Avian Dis..

[7] Gyuranecz M., Foster J.T., Dán Á., Ip H.S., Egstad K.F., Parker P.G., Higashiguchi J.M., Skinner M.A., Höfle U., Kreizinger Z. (2013). Worldwide Phylogenetic Relationship of Avian Poxviruses. J. Virol..

[8] van der Meer C.S., Paulino P.G., Jardim T.H.A., Senne N.A., Araujo T.R., dos Santos Juliano D., Massard C.L., Peixoto M.P., da Costa Angelo I., Santos H.A. (2022). Detection and molecular characterization of *Avipoxvirus* in *Culex* spp. (Culicidae) captured in domestic areas in Rio de Janeiro, Brazil. Sci. Rep..

[9] Tripathy D.N., Reed W.M. (2013). Chapter 10: Pox. Diseases of Poultry.

[10] Van Riper C., Van Riper S.G., Hansen W.R. (2002). Epizootiology and effect of avian pox on Hawaiian forest birds. Auk.

[11] Singh P., Kim T.J., Tripathy D.N. (2003). Identification and characterization of fowlpox virus strains using monoclonal antibodies. J. Vet. Diagn. Investig..

[12] da Silva P.S., Batinga Tde B., Sales T.S., Herval E.F.G., Ramos I., Maia P.C.C., Fernandes L.M.B. (2009). Fowlpox: Identification and adoption of prophylactic measures in backyard chickens in Bahia, Brazil. Rev. Bras. Cienc. Avic/Braz. J. Poult. Sci..

[13] Shrubsole-Cockwill A.N., Millins C., Jardine C., Kachur K., Parker D.L. (2010). Avian pox infection with secondary *Candida albicans* encephalitis in a juvenile golden eagle (*Aquila chrysaetos*). J. Avian Med. Surg..

[14] Reza K., Nasrin A., Mahmoud S. (2013). Clinical and pathological findings of concurrent poxvirus lesions and aspergillosis infection in canaries. Asian Pac. J. Trop. Biomed..

[15] Kleindorfer S., Dudaniec R.Y. (2006). Increasing prevalence of avian poxvirus in Darwin’s finches and its effect on male pairing success. J. Avian Biol..

[16] Silva R.A.F., Olinda R.G., Pimentel L.A., Maia L., Frade M.T.S., Kommers G.D., Galiza G.J.N., Dantas A.F.M. (2023). Cutaneous fungal infections secondary to avian pox in Northeast Brazil. Pesqui. Vet. Bras..

[17] Echenique J.V.Z., Bandarra P.M., Brauner R.K., Soares M.P., Coimbra M.A.A., Schild A.L. (2016). Infecção por pox vírus e *Aspergillus fumigatus* em *Bubo virginianus* (Coruja jacurutu). Pesqui. Vet. Bras..

[18] Smith K.F., Sax D.F., Lafferty K.D. (2006). Evidence for the role of infectious disease in species extinction and endangerment. Conserv. Biol..

[19] Wikelski M., Foufopoulos J., Vargas H., Snell H. (2004). Galápagos birds and diseases: Invasive pathogens as threats for island species. Ecol. Soc..

[20] Mori A., Baldaccini N.E., Baratti M., Caccamo C., Dessi-Fulghueri F., Grasso R., Pollonara E., Rodriguez F., Spena M.T., Giunchi D. Preliminary molecular investigation and characterization of subspecies in the Stone Curlew [*Burhinus oedicnemus*]. Proceedings of the 8th Conference of the European Ornithologists’ Union.

[21] Antor R.J., Margalida A., Heredia R., Madroño A., González C., Atienza J.C. (2005). Quebrantahuesos: *Gypaetus barbatus*. Libro Rojo de las Aves de España.

[22] López-Jiménez N., García de la Morena E., Bota G., Mañosa S., Morales M.B., Traba J. (2021). Sisón Comun, *Tetrax tetrax*. Libro Rojo de las Aves de España.

[23] Lierz M., Bergmann V., Isa G., Czerny C.P., Lueschow D., Mwanzia J., Prusas C., Hafez H.H. (2007). Avipoxvirus infection in a collection of captive stone curlews (*Burhinus oedicnemus*). J. Avian Med. Surg..

[24] Lecis R., Secci F., Antuofermo E., Nuvoli S., Scagliarini A., Pittau M., Alberti A. (2017). Multiple gene typing and phylogeny of avipoxvirus associated with cutaneous lesions in a stone curlew. Vet. Res. Commun..

[25] Calabuig P., Casal A.B., Camacho M., Orós J. (2011). Wildlife: Poxvirus infection in stone curlews in the Canary Islands. Vet. Rec..

[26] Burton E.J., Newnham R., Bailey S.J., Alexander L.G. (2014). Evaluation of a fast, objective tool for assessing body condition of budgerigars (*Melopsittacus undulatus*). J. Anim. Physiol. Anim. Nutr..

[27] Rae M.A. (2003). Practical avian necropsy. Semin. Avian Exot. Pet. Med..

[28] Source S.C., Medicine A., By P., Veterinarians A. (2006). Clinical Avian Medicine Book Review. J. Avian Med. Surg..

[29] Colom-Rivero A., Fernández A., Marrero-Ponce L., Castro-Alonso A., Rivero-Herrera C., Caballero-Hernández L., Sierra E. (2025). Molecular detection of a novel herpesvirus in the stone-curlew (*Burhinus oedicnemus*) from the Canary Islands. Avian Pathol..

[30] Baek H.E., Bandivadekar R.R., Pandit P., Mah M., Sehgal R.N.M., Tell L.A. (2020). TaqMan quantitative real-time PCR for detecting *Avipoxvirus* DNA in various sample types from hummingbirds. PLoS ONE.

[31] Lee L.H., Lee K.H. (1997). Application of the polymerase chain reaction for the diagnosis of fowl poxvirus infection. J. Virol. Methods.

[32] Kumar S., Stecher G., Suleski M., Sanderford M., Sharma S., Tamura K. (2024). MEGA12: Molecular Evolutionary Genetic Analysis Version 12 for Adaptive and Green Computing. Mol. Biol. Evol..

[33] Tamura K., Nei M., Kumar S. (2004). Prospects for inferring very large phylogenies by using the neighbor-joining method. Proc. Natl. Acad. Sci. USA.

[34] Pérez-Tris J., Williams R.A.J., Abel-Fernández E., Barreiro J., Conesa J.J., Figuerola J., Martinez-Martínez M., Ramírez A., Benitez L. (2011). A Multiplex PCR for Detection of Poxvirus and Papillomavirus in Cutaneous Warts from Live Birds and Museum Skins. Avian Dis. Dig..

[35] Felsenstein J. (1985). Confidence limits on phylogenies: An approach using the bootstrap. Evolution.

[36] Saitou N., Nei M. (1987). The neighbor-joining method: A new method for reconstructing phylogenetic trees. Mol. Biol. Evol..

[37] Gascuel O. (1997). BIONJ: An improved version of the NJ algorithm based on a simple model of sequence data. Mol. Biol. Evol..

[38] Le Loc’h G., Bertagnoli S., Ducatez M.F. (2015). Time scale evolution of avipoxviruses. Infect. Genet. Evol..

[39] Davidson W.R., Kellogg F.E., Doster G.L. (1980). An epornitic of avian pox in wild bobwhite quail. J. Wildl. Dis..

[40] Schoemaker N.J., Dorrestein G.M., Lumeij J.T. (1998). An avipoxvirus infection in a goshawk (*Accipiter gentilis*). Avian Pathol..

[41] Tell L.A. (2005). Aspergillosis in mammals and birds: Impact on veterinary medicine. Med. Mycol..

[42] Braga J.F.V., Couto R.M., Rodrigues M.C., Ecco R. (2020). *Avipoxvirus* detected in tumor-like lesions in a white-faced whistling duck (*Dendrocygna viduata*). Pesqui. Vet. Bras..

[43] Pesaro S., Biancani B., Fabbrizi G., Rossi G. (2009). Squamous cell carcinoma with presence of poxvirus-like inclusions in the foot of a pink-backed pelican (*Pelecanus rufescens*). Avian Pathol..

[44] Tsai S.S., Chang T.C., Yang S.F., Chi Y.C., Cher R.S., Chien M.S., Itakura C. (1997). Unusual lesions associated with avian poxvirus infection in rosy-faced lovebirds (*Agapornis roseicollis*). Avian Pathol..

[45] Van Riper C., Van Riper S.G., Goff M.L., Laird M. (1986). The Epizootiology and Ecological Significance of Malaria in Hawaiian Land Birds. Ecol. Monogr..

[46] Yeo G., Wang Y., Chong S.M., Humaidi M., Lim X.F., Mailepessov D., Chan S., How C.B., Lin Y.N., Huangfu T. (2019). Characterization of *Fowlpox* virus in chickens and bird-biting mosquitoes: A molecular approach to investigating Avipoxvirus transmission. J. Gen. Virol..

[47] Ruiz-Martínez J., Ferraguti M., Figuerola J., Martínez-De La Puente J., Williams R.A.J., Herrera-Dueñas A., Aguirre J.I., Soriguer R., Escudero C., Moens M.A.J. (2016). Prevalence and genetic diversity of avipoxvirus in house sparrows in Spain. PLoS ONE.

[48] Tompkins E.M., Anderson D.J., Pabilonia K.L., Huyvaert K.P. (2017). Avian pox discovered in the critically endangered waved albatross (*Phoebastria irrorata*) from the galápagos Islands, Ecuador. J. Wildl. Dis..

